# Complete Genome and Methylome Analysis of Psychrotrophic Bacterial Isolates from Lake Untersee in Antarctica

**DOI:** 10.1128/genomeA.01753-16

**Published:** 2017-03-16

**Authors:** Alexey Fomenkov, Vladimir N. Akimov, Lina V. Vasilyeva, Dale T. Andersen, Tamas Vincze, Richard J. Roberts

**Affiliations:** a New England BioLabs, Inc., Ipswich, Massachusetts, USA; b Institute of Biochemistry and Physiology of Microorganisms, Pushchino, Moscow Region, Russia; c S. N. Winogradsky Institute of Microbiology, Moscow, Russia; d Carl Sagan Center, SETI Institute, Mountain View, California, USA

## Abstract

This paper describes the complete genome sequences and methylome analysis of six psychrotrophic strains isolated from perennially ice-covered Lake Untersee in Antarctica.

## GENOME ANNOUNCEMENT

In this study, we performed a complete genome and methylome analysis of four bacterial isolates from concentrated water samples taken from a depth of 40 to 70 m in the aerobic zone of perennially ice-covered Lake Untersee (East Antarctica, 71°20′S 13°30′E) (1). The heterotrophic aerobic microbial cultures were isolated on LB agar plates under growth conditions of 8°C and 24°C for 1 to 2 weeks. DNA from the four isolated cultures, U13-I, U41, U17-1, and U14-5, were purified using a modified Qiagen method, and their genomes were sequenced using the Pacific Biosciences (PacBio) RSII sequencing platform. Briefly, SMRTbell libraries were constructed from a genomic DNA sample sheared to an average size of ~10 to 20 kb using the G-tubes protocol (Covaris, Woburn, MA, USA), end repaired, and ligated to hairpin adapters. Incompletely formed SMRTbell templates were digested with a combination of exonuclease III and exonuclease VII (New England BioLabs, Ipswich, MA, USA). Genomic DNA fragments and SMRTbell libraries qualification and quantification were performed using the Qubit fluorimeter (Invitrogen, Eugene, OR) and 2100 Bioanalyzer (Agilent Technologies, Santa Clara, CA). Four 20-kb SMRTbell libraries were prepared according to the modified PacBio sample preparation protocols, including additional separation on a BluePippin, and sequenced using C4 chemistry on 16 single-molecule real-time (SMRT) cells with a 180- to 240-min collection time. Sequencing reads were processed, mapped, and assembled by the Pacific Biosciences SMRT Analysis pipeline using the HGAP3 protocol and polished using Quiver (2) to yield six fully closed genomes, including nine plasmids (Table 1). The assembled sequences were annotated with Rapid Annotations using Subsystems Technology (RAST) (3) and the NCBI Prokaryotic Genome Annotation Pipeline (PGAP) and have been deposited at DDBJ/EMBL/GenBank.

**TABLE 1:** Summary of results

Isolate	Species	Genome size (bp)	Accession no.	Motif in genome^a^	Methylation type(s)	Assigned methylase(s)
U13-1	*Exiguobacterium* sp.	3,208,635	CP015731	CGGCSBYR^b^	m5C type II	ND^c^
				C**G**CCGVNY^b^	m5C type II	ND
U17-1	*Bacillus safensis*	3,741,806	CP015611	ND	ND	ND
U14-5	*B. safensis*	4,070,483	CP015607	C**C**WGG	m5C type II	M.Bsa145I
		58,536	CP015608			
		22,301	CP015609			
	*Roseomonas gilardi*	4,328,147	CP015583	GAACN_7_TCGC, GANTC	m6A type I, m6A type II	S.Rgi145I, M.Rgi145II
		702,334	CP015584			
		265,182	CP015585			
		64,339	CP015586			
		52,461	CP015587			
U41	*B. safensis*	3,735,180	CP015610	ND	ND	ND
	*Arthrobacter* sp.	4,386,370	CP015732	TTA**A**	m6A type II	M.AspU41I
		178,576	CP015733	GGC**A**N_6_**T**GA	m6A type I	S.AspU41III
		166,446	CP015734	GG**A**TCC	m6A type II	M.AspU41ORF21335P
		61,247	CP015735	C**C**WGG	m5C type I	M.AspU41II

^a^ Modified bases are highlighted in bold.

^b^ The m5C motif cannot be deduced unambiguously by the PacBio software. The motifs are unlikely to be correct as called.

^c^ ND, no motif detected, although the same m5C methylase gene is present in both strains.

The advantage of the PacBio sequencing platform is its ability to detect the epigenetic state of sequenced DNA, which allows for the identification of modified nucleotides and their corresponding motifs. Epigenetic modification at each nucleotide position was measured as kinetic variations (KVs) in the nucleotide incorporation rates, and methylated motifs were deduced from the KV data (4–6). Nine DNA methyltransferase recognition motifs were detected corresponding to N^6^-methyladenine (m6A) and probable 5-methylcytosine (m5C) modifications by direct SMRT sequencing. Two isolates, U14-5 and U41, each represented two organisms with different modification patterns. This allowed us to assign plasmids and chromosomes to the appropriate host based on their modification profiles. The motifs were then matched with methyltransferase genes, and the results are shown in Table 1. They have also been deposited in REBASE (7).

### Accession number(s).

Sequences have been deposited in GenBank under the accession numbers listed in Table 1.
